# Corrigendum to “Distinct cell death pathways induced by granzymes collectively protect against intestinal Salmonella infection” [Mucosal. Immun. 17 (2024) 1242–1255]

**DOI:** 10.1016/j.mucimm.2025.06.003

**Published:** 2025-08

**Authors:** Amanpreet Singh Chawla, Maud Vandereyken, Maykel Arias, Llipsy Santiago, Dina Dikovskaya, Chi Nguyen, Neema Skariah, Nicolas Wenner, Natasha B. Golovchenko, Sarah J. Thomson, Edna Ondari, Marcela Garzón-Tituaña, Christopher J. Anderson, Megan Bergkessel, Jay C.D. Hinton, Karen L. Edelblum, Julian Pardo, Mahima Swamy

**Affiliations:** a MRC Protein Phosphorylation and Ubiquitylation Unit, University of Dundee, Dundee DD1 5EH, United Kingdom; b Fundación Instituto de Investigación Sanitaria Aragón (IIS Aragón), Biomedical Research Centre of Aragón (CIBA), 50009 Zaragoza, Spain; c CIBER en Enfermedades Infecciosas, Madrid, Spain; d Departamento de Microbiología y Medicina Preventiva, Facultad de Medicina, Universidad de Zaragoza, Spain; e Department of Clinical Infection Microbiology & Immunology, Institute of Infection, Veterinary & Ecological Sciences, University of Liverpool, Liverpool, United Kingdom; f Biozentrum, University of Basel, Basel, Switzerland; g Department of Pathology, Molecular and Cell-Based Medicine, Icahn School of Medicine at Mount Sinai, New York, NY, USA; h Biological Services, School of Life Sciences, University of Dundee, Dundee DD1 5EH, United Kingdom; i Molecular Microbiology, School of Life Sciences, University of Dundee, Dundee DD1 5EH, United Kingdom; j Centre for Inflammation Research, Institute for Regeneration & Repair, University of Edinburgh, Edinburgh, United Kingdom

The authors regret that the article was published with the incorrect funding information. The authors want to remove the National Institutes of Health funding (NIH grants R01AI170617 and R01DK135272 to Karen L Edelblum) from the “Funding and acknowledgements” section because those grants have been recognized as non-essential for this study. The authors would like to apologize for any inconvenience caused.

