# Supplementary material for: Distinct cell death pathways induced by granzymes collectively protect against intestinal Salmonella infection
Source: Mucosal Immunol. 2024 Dec;17(6):1242–55. doi: 10.1016/j.mucimm.2024.08.006 (PMC11631773; doi:10.1016/j.mucimm.2024.08.006)
Supplement: Supplementary Data 1 [file mmc1.pdf]

Supplementary Information

Distinct cell death pathways induced by granzymes collectively protect against intestinal *Salmonella* infection

Amanpreet Singh Chawla, Maud Vandereyken, Maykel Arias, Llipsy Santiago, Dina Dikovskaya, Chi Nguyen, Neema Skariah, Nicolas Wenner, Natasha B. Golovchenko, Sarah J. Thomson, Edna Ondari, Marcela Garzón-Tituaña, Christopher J. Anderson, Megan Bergkessel, Jay C. D. Hinton, Karen L. Edelblum, Julian Pardo, Mahima Swamy

Graphical abstract

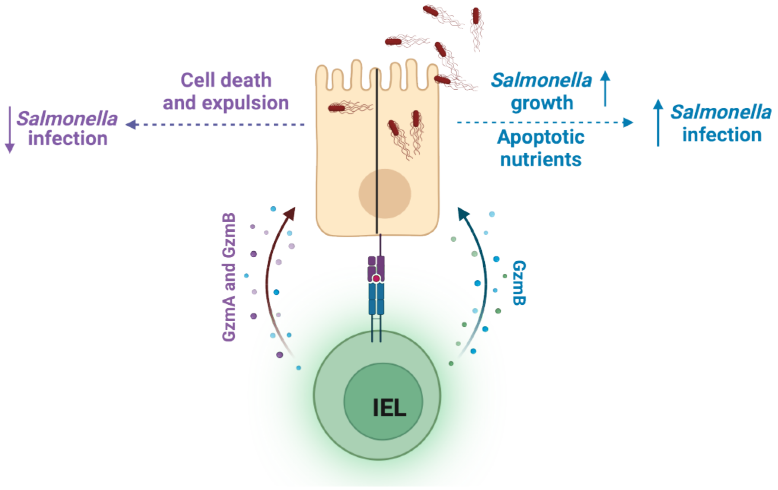

Supplementary Figure 1

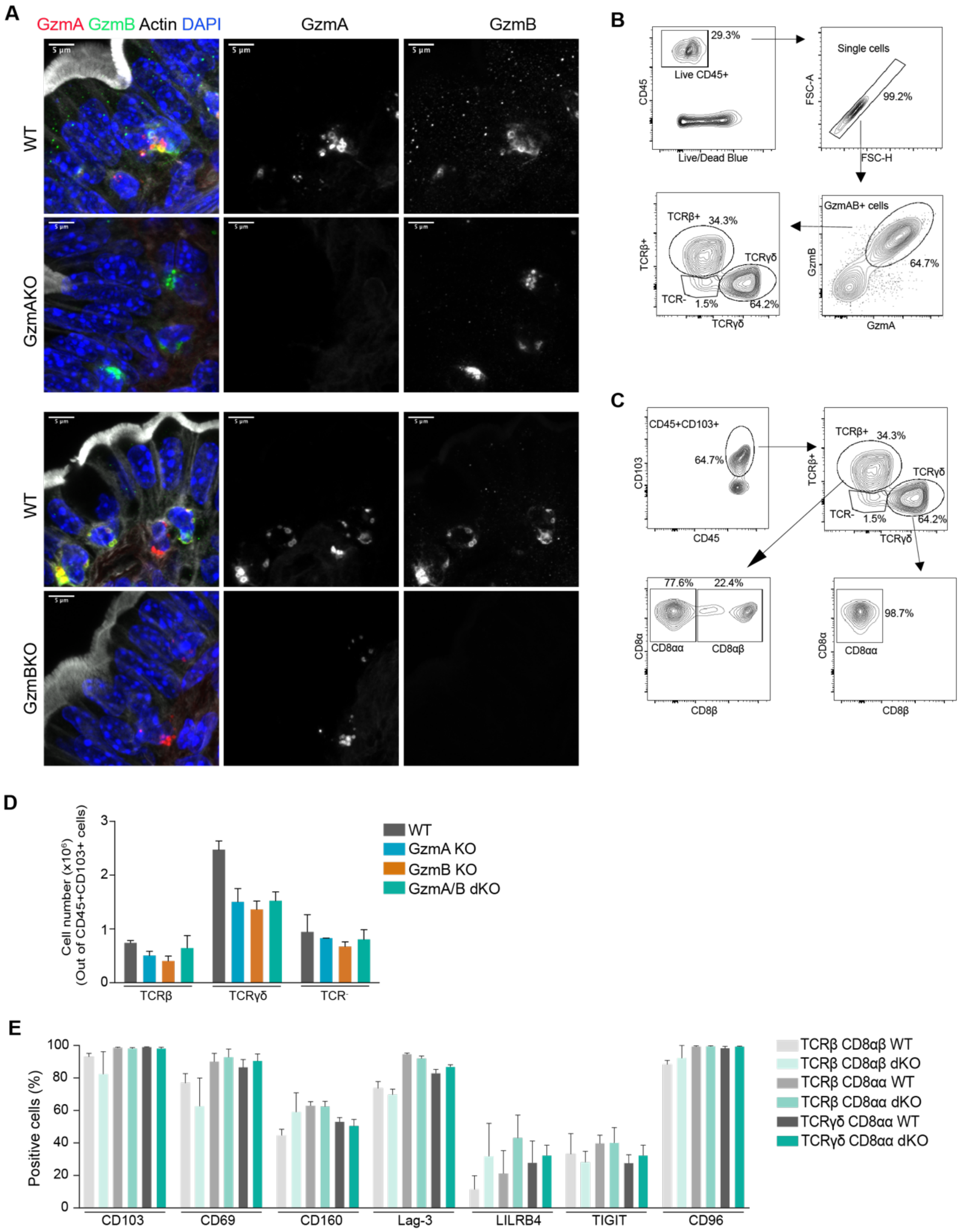

Supplementary Figure 1. **A.** Representative immunofluorescent micrographs to confirm the specificity of the GzmA and GzmB antibodies. Images show cells expressing GzmA (red) and GzmB (green) in the epithelial layer of a jejunal villus in WT, GzmA KO and GzmB KO. Sections were counterstained with phalloidin to show actin (white) and DAPI to show nuclei (blue on left panel). Scale bar=5μm. **B.** Gating strategy of GzmA/B$^+$ cells in the small intestine epithelium. Total CD45+ were gated as live, single, GzmAB$^+$ cells. These GzmA/B$^+$ cells can be further divided into TCRβ$^+$, TCRγδ$^+$ and TCR$^-$ subsets. **C.** Gating strategy of IEL in the small intestine - Total IEL were gated as live, single cell, CD45+CD103+ cells. CD45+CD103+ cells can be further subdivided into TCRβ$^+$ (CD8αα$^+$ and CD8αβ$^+$),

TCRγδ^{+} (CD8αα^{+} and CD8αβ^{+}) and TCR^{-} subsets. **D.** Absolute numbers of CD103^{+} IEL in WT littermate controls, GzmA KO, GzmB KO and GzmA/B dKO mice, with gating strategy shown in Suppl. Fig 1C **E.** Comparison of the percentage of positive WT and GzmA/B dKO IEL, from co-housed mice, for CD103, CD69, CD160, Lag-3, LILRB4, TIGIT and CD96 surface markers (n=3 each), with gating strategy shown in Suppl. Fig 1C. All data are presented as mean ± SEM. P values were calculated for (C-D) by ordinary one-way ANOVA with Sidak's multiple comparisons. Where no p-values are shown, no significance was found.

Supplementary Figure 2

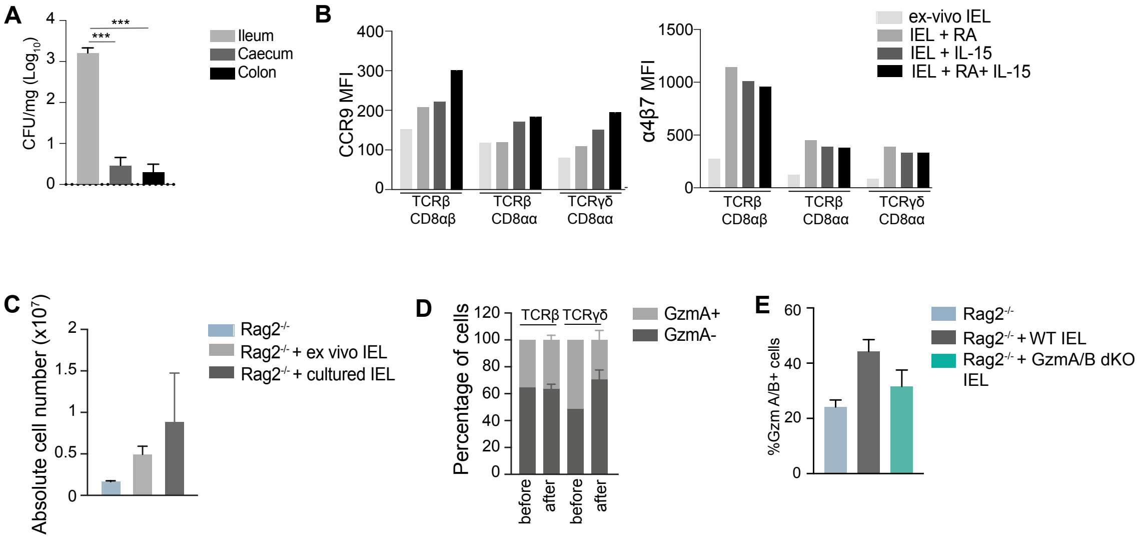

Supplementary Figure 2. **A.** Bar graph showing total bacterial (SL1344) counts in ileum, caecum, and colon of WT mice 4 days post infection (n=6). **B.** Comparison of the expression of CCR9 (left panel) and α4β7 (right) on ex vivo IEL and IEL cultured with retinoic acid and 100 ng/ml IL-15/Rα. **C.** Bar graph showing the number of $CD45^+$ cells collected in the SI epithelium after ex-vivo (n=3) or cultured (n=2) IEL transfer into $Rag2^{-/-}$ mice. **D.** Competitive transfer of cultured WT and GzmA/B dKO IEL (from co-housed mice) into $Rag2^{-/-}$ mice. WT and GzmA/B dKO IEL were mixed at a ratio of 1:1 before the transfer. Bar graphs show the ratio of GzmA positive cells in the TCRβ and TCRγδ populations, in the 1:1 mix before the transfer (n=2) and 4 weeks after the transfer, isolated from the adoptively transferred $Rag2^{-/-}$ mice (n=4). All data are presented as mean ± SEM. **E.** Percentages of $GzmA/B^+$ cells out of CD45+ cells isolated from the small intestinal epithelia of *Rag2*$^{-/-}$ mice, and from *Rag2*$^{-/-}$ mice that received IEL from either WT or GzmA/B dKO mice 4 weeks earlier, and that were then infected with SL1344. These data are from the mice shown in Fig. 2G-I. P values were calculated for (A) using ordinary one-way ANOVA with Sidak's multiple comparisons. *** $p<0.001$.

Supplementary Figure 3

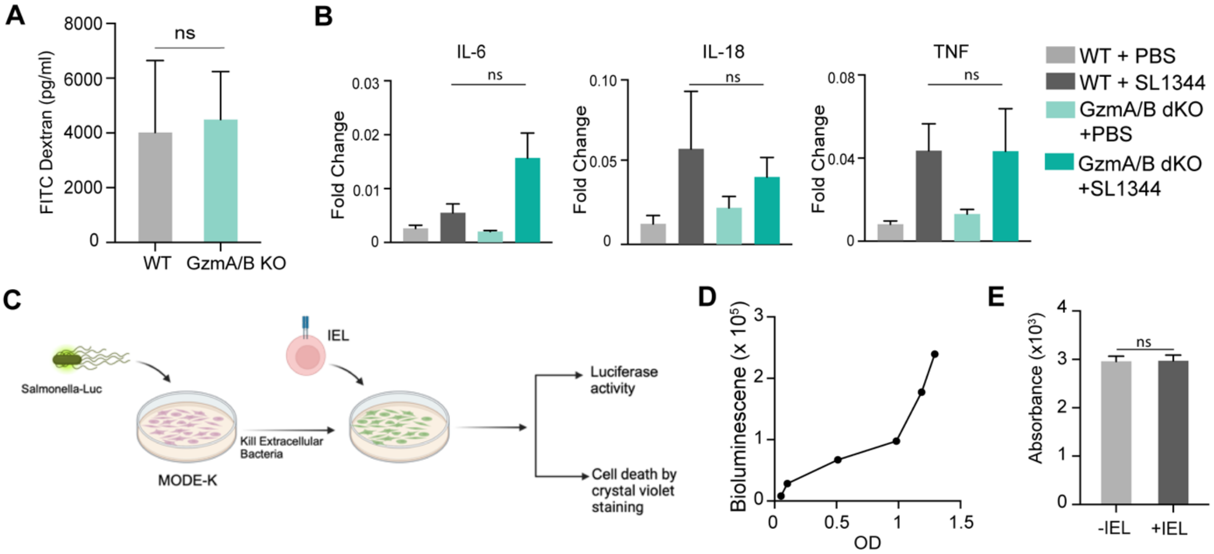

Supplementary Figure 3. **A.** Bar graphs comparing intestinal permeability in cohoused naïve WT and GzmA/B dKO mice (n=5/group). The concentration of FITC dextran was measured in the serum of the mice 4h after the gavage. **B.** Transcript fold change in the IL-6, IL-18 and TNF in small intestinal tissue of cohoused WT and GzmA/B dKO mice 3 days post *Salmonella* infection (n=3/group). **C.** Schematic showing the set-up of invitro MODE-K killing assay by IEL. MODE-K was infected with SL1344-lux for 0.5 h and then extracellular bacteria was killed using gentamycin. IEL were added to infected MODE-K and bioluminescence was acquired at different time points. **D.** Graph showing the correlation between bacterial OD and bacterial bioluminescence. **E.** IEL do not kill uninfected MODE-K. Bar graph showing the survival, measured using crystal violet method, of uninfected MODE-K in the presence or absence of WT IEL (n=3/group). All data are presented as mean ± SEM. P values were calculated for (A and E) using unpaired t-test and for (B) ordinary one-way ANOVA with Sidak's multiple comparisons. ns, not significant.

Supplementary Figure 4

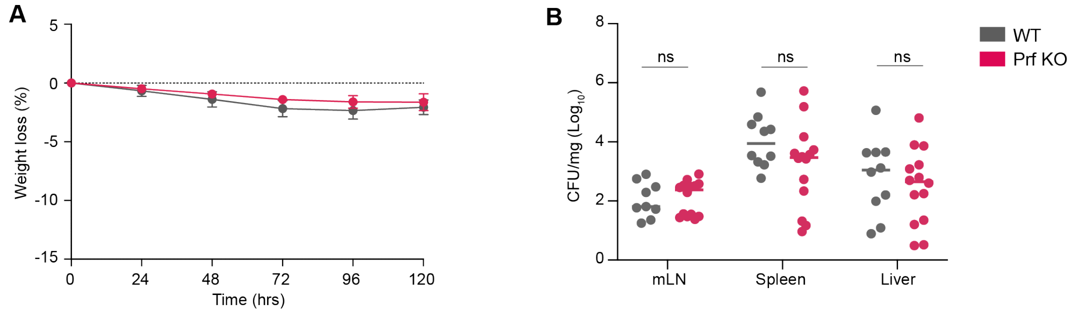

Supplementary Figure 4. Cohoused WT (n=10) and Prf1 KO (n=14) mice were orally infected with SL1344-GFP and culled 5dpi. Weight loss (**A**) and CFU/mg in MLN, spleen and liver at the time of sacrifice (**B**) are shown. Data were pooled from 2 independent experiments. All data are presented as mean ± SEM. P values were calculated for bacterial counts using the Mann-Whitney U-test and for all other comparisons, two-way ANOVA was used, with multiple comparisons using Sidak's multiple comparisons tests. ns: not significant.

Supplementary Figure 5

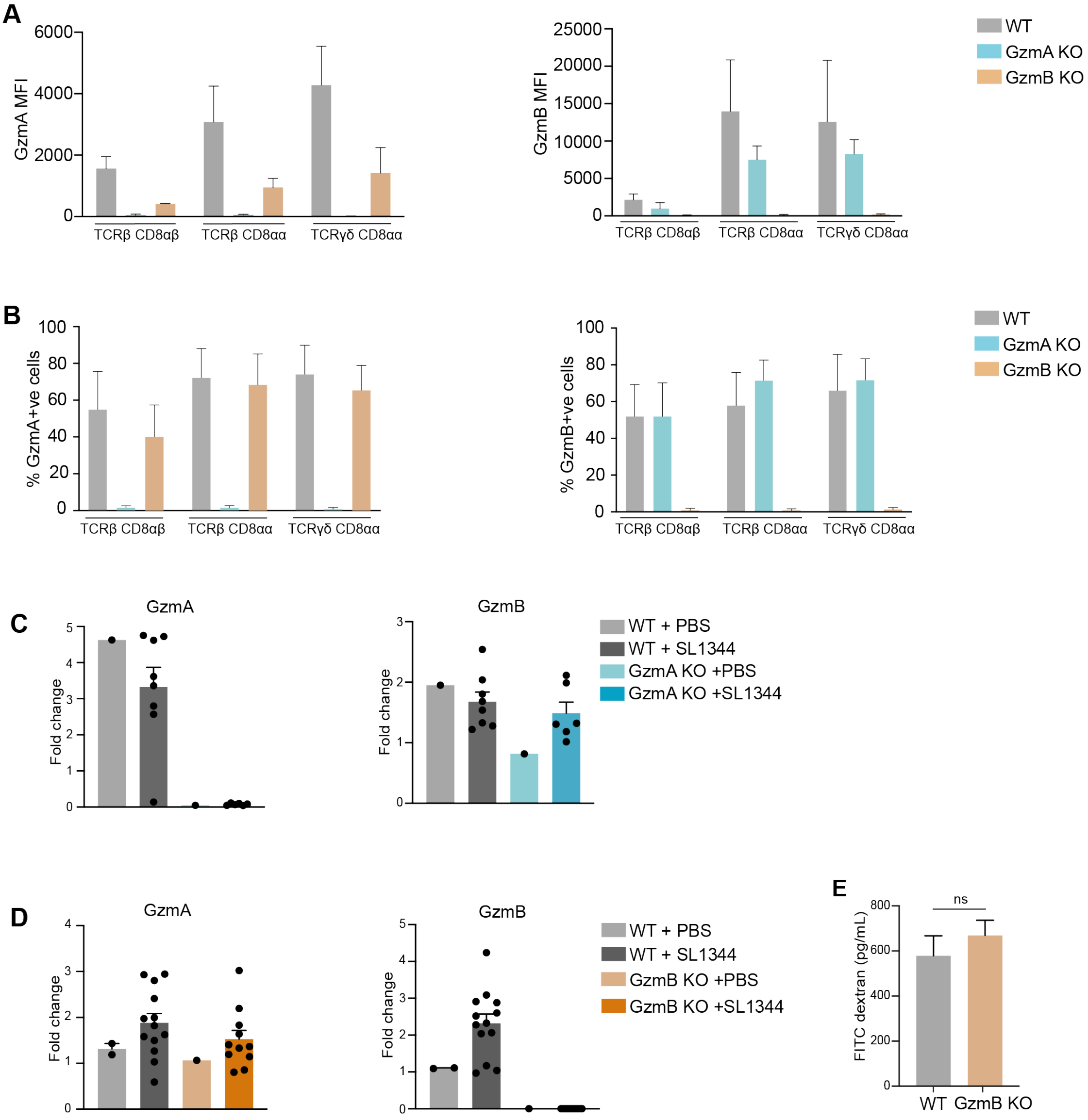

Supplementary Figure 5. A. Mean fluorescent intensity (MFI) of GzmA (left panel) and GzmB (right panel) in naïve GzmA KO (n=3), GzmB KO (n=2) compared to their WT littermate controls (n=3) mice. **B.** Comparison of the percentage of GzmA^{+} (left panel) or GzmB^{+} (right panel) IEL in GzmA KO, GzmB KO, and WT littermate controls (n>5/strain). **C-D.** Transcript fold change in *Gzma* and *Gzmb* in GzmA KO (C) and GzmB KO (D) as compared to WT littermate controls 5 days post *Salmonella* infection from mice in figure 4. **E.** Dot plots comparing intestinal permeability in naive GzmB KO and WT littermate control mice (n=5/group). The concentration of the FITC dextran was measured in the serum of the mice 4h after the gavage. Data were pooled from 2 independent experiments. All data are represented as mean ± SEM, except A where it is represented as mean ± SD. P-values were calculated for (B) by ordinary one-way ANOVA with Sidak's multiple comparisons, (C) by unpaired t-test. ns: not significant. Where no p-values are shown, no significance was found.

Supplementary Figure 6

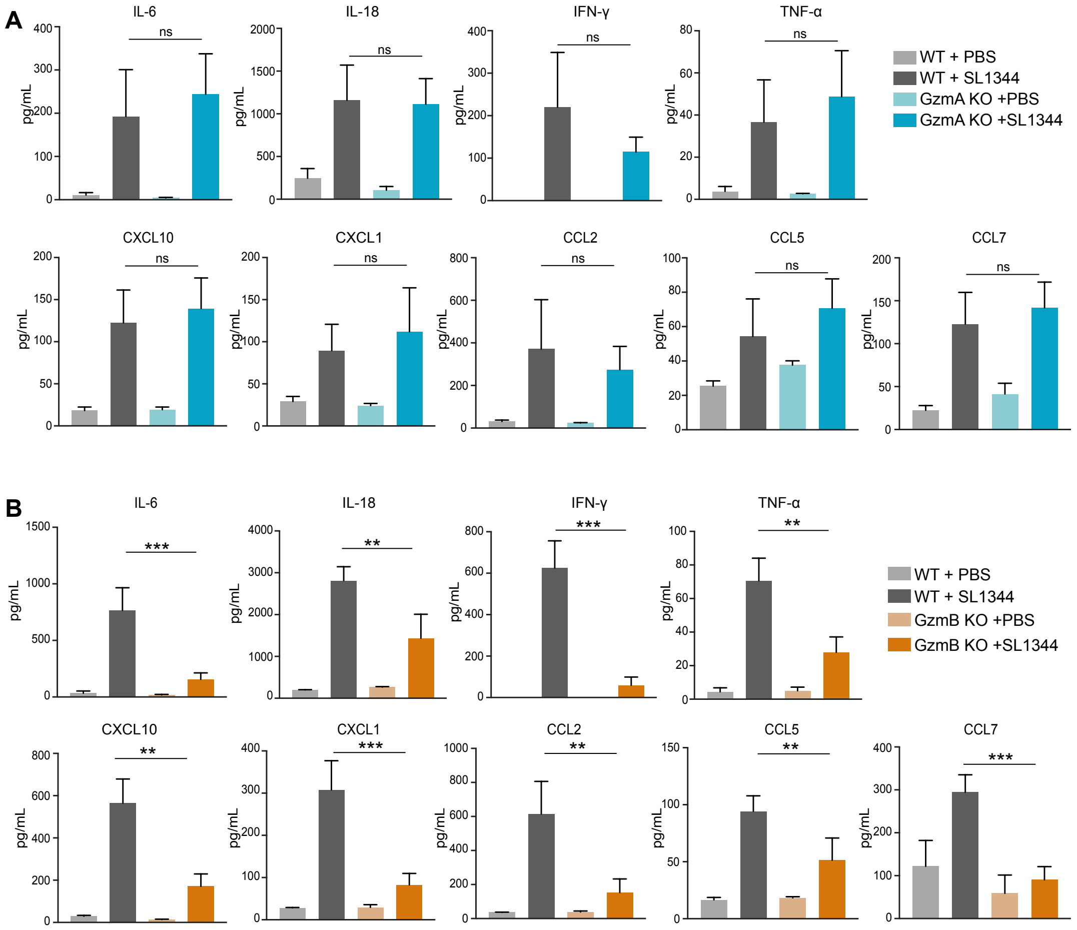

Supplementary Figure 6. Chemokine and cytokine levels in plasma of naïve and orally infected (**A**) GzmA sKO (n=12) and (**B**) GzmB sKO (n=12), compared to their WT littermate controls (n>9) mice from figure 4, 5 days post oral *Salmonella* infection. All data are represented as mean ± SEM. P-values were calculated for (A-B) by ordinary one-way ANOVA with Sidak's multiple comparisons, (C) by unpaired t-test. Standard annotations were used to denote significance: ns, not significant, ** p<0.01, *** p<0.001.

Supplementary videos 2 and 3. Representative time-lapse confocal microscopy videos of either (**2**) co-housed WT or (**3**) GzmA/B dKO IEL (in green) co-cultured with WT enteroids. Frames were acquired every 2.5 minutes for 90 min.
